# Factor Structure of the Affective Style Questionnaire in Flemish Adolescents

**DOI:** 10.5334/pb.369

**Published:** 2017-07-03

**Authors:** Sara Erreygers, Pieter Spooren

**Affiliations:** 1 University of Antwerp, BE

**Keywords:** emotion regulation, affective style, concealing, adjusting, tolerating, exploratory structural equations modeling

## Abstract

Emotion regulation plays an important role in both healthy and problematic adolescent psychological functioning. Emotion regulation tendencies can be assessed with the Affective Style Questionnaire (ASQ; Hofmann & Kashdan, 2010), but its validity in Dutch speaking adolescents has not been investigated so far. Two methods, namely traditional confirmatory factor analysis (CFA) and the recently developed exploratory structural equations modeling (ESEM), were compared to examine the dimensional structure of the ASQ in a Flemish adolescent sample (*N* = 1,601). Although, as expected, the ESEM-model fit the data better than the CFA-model, the fit indices indicated that both models did not have an acceptable fit. With a shortened version of the ASQ, model fit improved substantially, but only the ESEM solution provided a good fit. The ESEM results support the use of the adapted ASQ to effectively assess the affective styles of concealing, adjusting and tolerating in Dutch-speaking adolescents.

Adolescence is a developmental phase characterized by challenges across multiple life domains, associated with biological, psychological, intellectual and social changes (e.g., puberty, increasing importance of peers over parents, changing nature of relationships). These challenges expose adolescents to stress and evoke multiple emotions (Rosenblum & Lewis, 2003). Adolescents not only experience more intense emotions than adults, they also experience negative emotions more frequently than younger and older age groups (Larson, Csikszentmihalyi, & Graef, 1980; Rosenblum & Lewis, 2003). How adolescents cope with their emotions, *emotion regulation*, is an important predictor and indication of psychosocial functioning (Zeman, Cassano, Perry-Parrish, & Stegall, 2006). Emotion regulation has been defined as “the extrinsic and intrinsic processes responsible for monitoring, evaluating, and modifying emotional reactions, especially their intensive and temporal features, to accomplish one’s goals” (Thompson, 1994, pp. 2–3). Research has shown that successful emotion regulation is associated with positive outcomes (e.g., John & Gross, 2004) whereas difficulties with emotion regulation are related to reduced social competence and psychological functioning (e.g., Hofmann, Sawyer, Fang, & Asnaani, 2012; Zeman et al., 2006).

Because emotion regulation plays such an important role in adaptive psychosocial functioning in adolescence, measures that assess individual differences in emotion regulation can provide crucial information about adolescent emotional development. Individual differences in the tendencies to react to and regulate emotions have been referred to as differences in affective styles (Davidson, 1998; Hofmann et al., 2012). Three different affective styles are commonly distinguished, based on research on Gross’s (1998) process model of emotion regulation: concealing, adjusting, and tolerating (Hofmann et al., 2012). Concealing involves response-focused strategies such as suppression, which are intended to hide and avoid emotions after they arise. Adjusting encompasses the modulation of emotions as needed in a particular context by balancing and readjusting emotional experience and expression. Tolerating refers to an accepting and non-defensive reaction towards strong and arousing emotions.

Hofmann and Kashdan (2010) have developed the Affective Style Questionnaire (ASQ) to assess individual differences in the aforementioned affective styles. In two studies with US undergraduate university students, their instrument demonstrated excellent convergent and discriminant validity and reliability. The instrument has also successfully been used with adolescents (Lougheed & Hollenstein, 2012). Recently, the ASQ has been evaluated in German and Japanese (adult) student samples. In the German study, the original factor structure was replicated and the scale showed good psychometric properties (Graser et al., 2012). In the Japanese study, four instead of three factors were found in a factor analysis: the three original factors (adjusting, concealing and tolerating) plus an extra factor which the authors labelled *holding* (Ito & Hofmann, 2014). This fourth factor reflected the ability to keep emotions under control via self-restraint. The subscales had acceptable convergent and discriminant validity, but the reliability of the tolerating subscale was poor (Cronbach’s alpha below .50).

Importantly, the German and Japanese study used different methods to evaluate the factor structure of the instrument. The Japanese study employed exploratory (EFA) and confirmatory factor analyses (CFA), methods which are commonly used to examine and replicate the factor structure of a scale. The CFA measurement model is restrictive: It specifies a simple structure in which each indicator is influenced by only one factor, which means that no cross-loadings are allowed (i.e., non-target loadings are fixed to zero). In contrast, the German study used a more recent approach to evaluate the ASQ factor structure: exploratory structural equation modeling (ESEM). This approach combines CFA and EFA measurement model parts and is less restrictive than CFA (that is, it allows small cross-loadings that are well motivated by the theory), generally resulting in better-fitting models (Asparouhov, Muthén, & Muthen, 2009; Marsh et al., 2009). In CFA, the small model misspecifications created by fixing all cross-loadings to zero can have a large influence on the rest of the model (e.g., biased parameter estimates) (Asparouhov et al., 2009). Small cross-loadings are not deleted in ESEM, resulting in models that fit better and conform better with theory.

To the best of our knowledge, so far there is no scale available to assess affective styles in Dutch. However, there is a Dutch instrument available to measure emotion regulation styles in children and adolescents, the FEEL-KJ (Braet, Cracco, & Theuwis, 2013). This instrument focuses on the specific regulation of anger, sadness, and fear, and distinguishes between 15 adaptive, maladaptive, and external regulation strategies. Which strategies are categorized as adaptive and maladaptive was based on factor analysis rather than on theoretical grounds (Grob & Smolenski, 2005). In contrast, the ASQ distinguishes three more general adaptive styles, based on theory and factor analysis. These affective styles are not emotion-specific, but are seen as habitual tendencies to regulate negative emotions across situations. Moreover, the ASQ has the advantage of being a short instrument (only 20 items), compared to the FEEL-KJ (90 items), which makes it easier to use in multi-instrument studies. As the ASQ seems a promising instrument to assess affective styles, we aimed to examine its factor structure in a Dutch-speaking adolescent sample. We used both CFA and ESEM to compare their outcomes on the dimensional structure of the ASQ.

## Method

### Participants

In total 1,601 Dutch-speaking adolescents participated. Participants were in the first year of Belgian secondary education and had a mean age of 13.2 years (*SD* = .4, range: 10–15). Forty-six percent of the students were boys and 54.0% girls. 90.6% of the students were in the general education track (1A), 9.4% in vocational education (1B). A large majority (94.5%) of them spoke Dutch with one or both of their parents at home; 24.7% of the participants (also) speak another language at home.

### Procedure

The data were collected in the context of a broader longitudinal study. Data collection took place during the first wave of this study in March to May 2015. Participants were recruited through their schools. Schools were randomly selected from one province in Flanders (the Dutch-speaking part of Belgium). Of the 29 schools that were contacted, 13 agreed to participate. Prior to administration, informed consent was obtained from the students, their parents and the school principal; all except 13 students agreed to participate.

The data were collected through pen-and-paper and electronic surveys, which were administered to the students in classrooms during school hours. In most schools, the author was present during the administration of the survey to answer questions. A few schools preferred to administer the questionnaire by their own personnel during spare hours. The study received approval by the Ethics Committee for the Social Sciences and Humanities of the University of Antwerp (reference number SHW_14_39_02).

### Measures

Affective styles were assessed by the ASQ (Hofmann & Kashdan, 2010; see Table 1 for scale items). Participants answered 20 statements about their tendency to react to emotions on a 5-point Likert-type scale that ranges from 1 (*not true of me at all*) to 5 (*extremely true of me*). Factor analyses of the original scale revealed three factors: Adjusting, Concealing, and Tolerating.

**Table 1:** ESEM and CFA Factor Solutions of 20-Item ASQ.

	Factor loadings		
	
Items	Concealing	Adjusting	Tolerating

Concealing			
C1 People usually can’t tell how I am feeling inside.	0.439/0.188	–0.301	0.040
C2 I often suppress my emotional reactions to things.	0.352/0.395	–0.092	0.266
C3 I am good at hiding my feelings.	0.732/0.677	0.066	–0.023
C4 People usually can’t tell when I am upset.	0.605/0.541	–0.036	0.042
C5 People usually can’t tell when I am sad.	0.616/0.572	0.032	–0.010
C6 I can act in a way that people don’t see me being upset.	0.635/0.708	0.115	0.069
C7 I could easily fake emotions.	0.403/0.552	0.189	0.057
C8 I can hide my anger well if I have to.	0.347/0.680	0.433	0.001
Adjusting			
A1 I have my emotions well under control.	0.280	0.332/0.639	0.257
A2 I can avoid getting upset by taking a different perspective on things.	0.308	0.115/0.544	0.375
A3 I am able to let go of my feelings.	–0.181	0.175/0.363	0.501
A4 I can calm down very quickly.	0.150	0.574/0.693	0.149
A6 I know exactly what to do to get myself into a better mood.	0.000	0.723/0.719	0.083
A7 I can get into a better mood quite easily.	–0.082	0.883/0.782	0.057
Tolerating			
T1 I can tolerate having strong emotions.	0.343	0.353	0.227/0.694
T2 It’s ok if people see me being upset.	–0.149	–0.026	0.525/0.237
T3 It’s ok to feel negative emotions at times.	0.044	–0.109	0.645/0.372
T4 I can tolerate being upset.	0.191	0.384	0.339/0.692
T5 There is nothing wrong with feeling very emotional.	–0.131	0.008	0.622/0.339

*Note.* Factor loadings of the ESEM solution appear before the slash and factor loadings of the ICM-CFA solution after the slash. All parameters are completely standardized. The ICM-CFA model has an independent cluster structure in which each of the ASQ items is allowed to load on only one single latent factor and all non-target loadings are constrained to be zero. For clarity, these ICM-CFA non-target zero loadings are not displayed.

A Dutch version of the questionnaire was created through the back-translation method (Brislin, 1970). First, the first author, who is fluent in English, translated the ASQ into Dutch. Then, a professional translator translated the Dutch version back into English. The author compared this version with the original ASQ and adjusted some of the wording of the Dutch items to better align with the original version. Finally, a bilingual colleague translated the modified Dutch version to English and suggested some additional minor adjustments to the Dutch wording.

### Data Analysis

We compared two factor analytical approaches: CFA and ESEM. In an independent clusters model-CFA (ICM-CFA), each item is allowed to load on one factor, in this case the respective subscale, and all other factor loadings are constrained to be zero. This procedure was used in the Japanese study (Ito & Hofmann, 2014). However, Asparouhov, Muthén, and Muthén (2009) and Marsh and colleagues (2009) have argued that because of its use of a strict measurement model, CFA has a number of disadvantages. For instance, CFA specifies zero cross-loadings, whereas measurement instruments often have small cross-loadings that are theoretically meaningful. By constraining all cross-loadings to zero, the measurement model often becomes too parsimonious for the data. This may lead to an ill-fitting model, which, in many cases, requires a number of model modifications to arrive at a good fit. Additionally, misspecification of the zero cross-loadings often leads to distorted factors, resulting in inflated factor correlations. With regards to the ASQ, there are theoretical reasons to expect cross-loadings of a few items. For instance, the item “I can hide my anger well if I have to” is a concealing strategy, but it could also be seen as an instance of adjusting to the situation, as in particular situations, it is not appropriate or constructive to show one’s anger. Additionally, “I am able to let go of my feelings”, which is supposed to load on *adjusting*, might also load on *tolerating*, as it reflects the ability to first acknowledge and accept emotions, and then to move on.

The recent ESEM approach provides a good alternative to the traditional CFA. ESEM integrates EFA and CFA by using EFA factor loadings matrix rotations in combination with SEM parameters and tests of model fit. This approach was used in the German study (Graser et al., 2012) and, contrary to the Japanese CFA model, the ESEM model succeeded to replicate the original ASQ factor structure.

The data were analyzed in Mplus 6.11 (Muthén & Muthén, 2015). Because the ASQ variables are ordinal and not normally distributed, we used the weighted least squares means and variances adjusted (WLSMV) estimator (Sass, Schmitt, & Marsh, 2014). In the ESEM models we used an oblique Geomin rotation with an epsilon value of 0.5 (cf. Marsh et al., 2009; Vazsonyi, Ksinan, Mikuška, & Jiskrova, 2015). To determine how well the models fit the data, several goodness-of-fit indices were examined, including the root mean square error of approximation (RMSEA, acceptable threshold < 0.07), the Comparative Fit Index (CFI, acceptable threshold > 0.95), and the Tucker-Lewis Index (TLI, acceptable threshold > 0.95) (Hooper, Coughlan, & Mullen, 2008). All factor loadings were fully standardized.

## Results

First, we performed an ICM-CFA of the translated version of the ASQ, following the factor structure of the original study (Hofmann & Kashdan, 2010). Examining the goodness-of-fit statistics (Table 2), this model did not fit the data well: χ^2^(167) = 3992.361, *p* < 0.001; CFI = 0.761; TLI = 0.728; RMSEA = 0.120 [0.116, 0.123]. All the factor loadings were significant and most were quite high (Table 1); non-target loadings were constrained to be zero. The factor correlations were high and significant (Table 3), ranging from 0.57 between Adjusting and Concealing to 0.93 between Tolerating and Adjusting. We also performed an ICM-CFA of the Japanese four-factor model (Ito & Hofmann, 2014), which resulted in slightly better fit statistics than the three-factor model, but the overall model fit was still poor: χ^2^(98) = 1626.025, *p* < 0.001; CFI = 0.849; TLI = 0.815; RMSEA = 0.099 [0.094, 0.103]. When CFA-models fit the data this poorly, it is common practice to examine the modification indices and to relax the restrictive CFA-constraints by allowing for cross-loadings and item correlations. However, in this case a large number of modifications would have had to be made to arrive at an acceptable fit, with considerable deviations from the original measurement model. Moreover, the issue of the inflated correlations would probably still remain present (Marsh et al., 2009).

**Table 2:** Summary of Goodness-of-Fit Statistics for the Models Tested.

Scale	Model	χ^2^	*df*	χ^2^/*df*	*p*(χ^2^)	CFI	TLI	RMSEA	90% CI RMSEA

20-Item ASQ	ICM-CFA	3992.361	167	23.906	0.000	0.761	0.728	0.120	[0.116, 0.123]
20-Item ASQ	ESEM	1930.925	133	14.518	0.000	0.888	0.840	0.092	[0.088, 0.096]
16-Item ASQ	ICM-CFA	1724.249	101	17.072	0.000	0.860	0.834	0.100	[0.096, 0.104]
16-Item ASQ	ESEM	555.691	75	7.409	0.000	0.959	0.934	0.063	[0.058, 0.068]
16-Item ASQ	ESEM MI	767.954	240	3.200	0.000	0.954	0.954	0.053	[0.048, 0.057]

*Note.* ICM-CFA = independent clusters model – confirmatory factor analysis; ESEM = exploratory structural equations modeling; MI = measurement invariance across genders; CFI = comparative fit index; TLI = Tucker-Lewis Index; RMSEA = root mean square error of approximation; 90% CI RMSEA = 90 percent confidence interval RMSEA.

**Table 3:** ESEM (and ICM-CFA) Factor Correlations for the 20-Item and 16-Item ASQ.

	Factor correlations		
	
	20-Item ASQ		
	
Factor	Concealing	Adjusting	Tolerating

Concealing	1.00/1.00	0.57/0.54	0.60/0.20
Adjusting	0.25/0.22	1.00/1.00	0.93/0.39
Tolerating	0.18/0.14	0.33/0.26	1.00/1.00

*Note.* ICM-CFA factor correlations appear above the diagonal, ESEM factor correlations below the diagonal. Numbers before the slash indicate factor correlations of the 20-item ASQ, numbers after the slash of the 16-item ASQ.

Compared to the ICM-CFA model, the three-factor ESEM model fit the data better, although model fit was still not acceptable (Table 2): χ^2^(133) = 1930.925, *p* < 0.001; CFI = 0.888; TLI = 0.840; RMSEA = 0.092 [0.088, 0.096]. Furthermore, the correlations between the factors were substantially lower in the ESEM model than the CFA model, ranging from 0.18 to 0.33. Most of the factor loadings were moderate to high, although a few items had a low loading on their designated factor but a high loading on another factor (Table 1).

Taken together, these results suggested that the ESEM model fit the data better than the ICM-CFA model. Nonetheless, both models did not have an acceptable fit of the data. Thus, the original factor structure of the ASQ could not be adequately replicated in our Flemish adolescent sample. Because affective styles are such an important concept related to adolescent functioning and there are no other validated Dutch instruments available to measure them, we decided to adapt the scale with the aim of achieving an acceptable model fit. First, during the administration of the survey in schools, the author had noted that some items were hard to understand for the students, as students repeatedly asked questions about their meaning. For example, the wording of items T1 and T4 was difficult to grasp for many students (they did not understand the meaning of ‘*verdragen*’, the translation of *tolerating*). In addition, the adolescents had trouble understanding what was meant by items A2 and A3. This might explain why these items had low factor loadings on their own factor and high cross-loadings. These items were deleted from analysis.

With this shortened, 16-item version of the ASQ, we performed another ICM-CFA and ESEM analysis. The 16 items and their factor loadings for both models are displayed in Table 4. Inspection of the ESEM factor loadings (Table 4) reveals that all items loaded on their own factor (>minimum loading of 0.32, cf. Costello & Osborne, 2005) and had only small factor loadings on the other factors, approximating a simple structure. Only one item (item C8: “I can hide my anger well if I have to”) cross-loaded on another factor. This item originally belonged to the Concealing subscale, but also loaded on the Adjusting subscale. However, this can be explained when considering the meaning of the item. Hiding anger if you have to can not only be seen as a tendency to conceal anger, but it can also be an adaptive response to situations where it is not appropriate to express angriness. Thus, the cross-loading on Adjusting is theoretically relevant and meaningful.

**Table 4:** ESEM and CFA Factor Solutions of 16-Item ASQ.

	Factor loadings		
	
Items	Concealing	Adjusting	Tolerating

Concealing			
C1 People usually can’t tell how I am feeling inside.	0.444/0.224	–0.295	0.084
C2 I often suppress my emotional reactions to things.	0.327/0.353	–0.039	0.216
C3 I am good at hiding my feelings.	0.713/0.678	0.090	–0.027
C4 People usually can’t tell when I am upset.	0.622/0.561	–0.024	0.056
C5 People usually can’t tell when I am sad.	0.626/0.590	0.033	0.015
C6 I can act in a way that people don’t see me being upset.	0.647/0.715	0.127	0.102
C7 I could easily fake emotions.	0.418/0.543	0.205	0.039
C8 I can hide my anger well if I have to.	0.354/0.665	0.431	0.037
Adjusting			
A1 I have my emotions well under control.	0.213	0.341/0.522	0.146
A4 I can calm down very quickly.	0.157	0.576/0.677	0.093
A5 I can get out of a bad mood very quickly.	0.093	0.611/0.713	0.159
A6 I know exactly what to do to get myself into a better mood.	0.022	0.722/0.761	0.108
A7 I can get into a better mood quite easily.	–0.060	0.886/0.838	0.092
Tolerating			
T2 It’s ok if people see me being upset.	–0.137	0.029	0.441/0.385
T3 It’s ok to feel negative emotions at times.	0.085	–0.052	0.594/0.644
T5 There is nothing wrong with feeling very emotional.	–0.199	0.015	0.748/0.693

*Note.* Factor loadings of the ESEM solution appear before the slash and factor loadings of the ICM-CFA solution after the slash. All parameters are completely standardized. The ICM-CFA model has an independent cluster structure in which each of the ASQ items is allowed to load on only one single latent factor and all non-target loadings are constrained to be zero. For clarity, these ICM-CFA non-target zero loadings are not displayed.

Both analytical approaches resulted in a much better fit with the 16-item ASQ than with the 20-item ASQ (Table 2). Furthermore, comparing the ICM-CFA and ESEM model, the ESEM model again fit the data better than the ICM-CFA model. The model fit indices (Table 2) suggested that the ESEM model of the 16-item ASQ had an acceptable fit (CFI > 0.95, RMSEA < 0.07) whereas the ICM-CFA model had not (CFI < 0.95, RMSEA > 0.07). Furthermore, the correlations between the factors were reduced for the 16-item ASQ compared to the 20-item ASQ (Table 3), although the factor correlations were still higher in the ICM-CFA (range: 0.20–0.54) than in the ESEM solution (range: 0.14–0.26).

To examine whether the 16-item ASQ demonstrated measurement equivalence across gender groups, a measurement invariance ESEM model with equality of the factor variances and covariances, in addition to measurement invariance of the intercepts and factor loading matrices was tested. The fit of this model with constraints across gender groups was good (CFA > 0.95, RMSEA < 0.07; see Table 2), suggesting that boys and girls interpret the ASQ items in a similar manner. Nevertheless, there were significant differences between boys and girls on the mean scores of each subscale: Boys scored higher on Concealing (*M*_boys_ = 2.81, *M*_girls_ = 2.69, *t*(1511,23) = 3.09, *p* = .002) and Adjusting (*M*_boys_ = 3.30, *M*_girls_ = 3.08, *t*(1511,28) = 4.76, *p* = .000), and lower on Tolerating (*M*_boys_ = 2.76, *M*_girls_ = 2.85, *t*(1571) = 2.16, *p* = .035) than girls. These differences replicate the gender differences reported in the German study (Graser et al., 2012).

Finally, we examined the internal consistency of the subscales by calculating Cronbach’s alpha for each of the subscales, first with the original ASQ items and then with the shortened version. For both versions, internal consistency was acceptable for the Concealing, *α* = 0.74, and Adjusting, *α* = 0.77 – 0.79, subscales, but poor for the Tolerating subscale, *α* = 0.56 – 0.59.

## Discussion and Conclusion

Emotion regulation tendencies (or affective styles) have an important influence on adaptive psychological functioning in adolescence (e.g., Zeman et al., 2006). However, to date there was no validated Dutch instrument available to measure affective styles in adolescents. Our study suggests that an adapted version of the ASQ (Hofmann & Kashdan, 2010) can effectively be used to assess the affective styles of concealing, adjusting and tolerating in Dutch-speaking adolescents using the ESEM approach.

Our results showed that the ESEM approach resulted in a better model fit than ICM-CFA. However, model fit for the original, 20-item ASQ was not acceptable, even when using ESEM. Therefore, we omitted four items that were poorly understood by the adolescents in our sample and re-validated the factor structure of this shortened version of the ASQ. The ESEM and ICM-CFA model had a substantially better fit for the 16-item ASQ, but only the ESEM approach resulted in an acceptable fit. Moreover, ESEM factor correlations were lower than ICM-CFA factor correlations. The restrictive CFA measurement model constrains all cross-loadings to be zero, which usually leads to inflated factor correlations (Asparouhov et al., 2009). Our study is in line with other studies that show how ESEM leads to lower factor correlations and more appropriate parameter estimates (Marsh, Liem, Martin, Morin, & Nagengast, 2011).

Using the 16-item adapted ASQ, we were able to replicate the factor structure of the original instrument. However, in the original study (Hofmann & Kashdan, 2010), all the items of the scale loaded on one factor only, whereas in our study one item (C8: “I can hide my anger well if I have to”) of the adapted scale loaded on two factors, Concealing and Adjusting. Considering the meaning of this item, this cross-loading is theoretically relevant, as this emotion regulation strategy can aim to conceal the emotion as well as to adjust oneself to contextual demands (when it is not socially acceptable to show anger). This again illustrates the added value of ESEM compared to ICM-CFA, where cross-loadings are constrained to be zero and such meaningful double loadings are not allowed.

The results of this study should be interpreted in light of some limitations. First, the internal consistency of the Tolerating subscale was poor. Practitioners who use this subscale should interpret the results with caution. Second, we did not examine the convergent and divergent validity of the ASQ in our sample. Although the dimensional structure of the adapted ASQ appears to be good, we did not explore how the subscales relate to other instruments measuring similar constructs and how the subscales differentially relate to other constructs. This study was part of a larger project assessing several diverse constructs, and due to time and space constraints, unfortunately we were not able to include extra instruments measuring emotion regulation to evaluate the external validity of the ASQ. However, the analysis of the measurement invariance does support the validity of the instrument across gender groups. Third, the factorial validity of the adapted ASQ should be tested in other samples (e.g., older participants, people from the Netherlands) to generalize the results. Fourth, we did not take into account the multilevel structure of our data (students nested in classrooms and schools). However, to date it is only possible to perform multilevel CFA analyses (and not multilevel ESEM), which impedes straightforward comparisons between the two approaches.

Despite these limitations, this study demonstrates the factorial validity of the Dutch 16-item version of the ASQ using the recently developed ESEM approach. This scale can effectively be used to assess emotion regulation tendencies in Dutch-speaking adolescents, and can thereby provide valuable insights into their abilities to adaptively cope with their emotions during a life phase in which emotions are especially intensive and variable.

## Additional File

The additional file for this article can be found as follows:

10.5334/pb.369.s1 Appendix 16-item Affective Style Questionnaire, Dutch translation.

## Author Information

This research was funded by a grant from the Research Foundation Flanders, grant number FWO G.0335.14 N.
