# Supplementary material for: Factor Structure of the Affective Style Questionnaire in Flemish Adolescents
Source: Psychol Belg. 2017 Jul 3;57(2):112–22. doi: 10.5334/pb.369 (PMC6194515; doi:10.5334/pb.369)
Supplement: Appendix — 16-item Affective Style Questionnaire, Dutch translation. [file pb-57-2-369-s1.pdf]

Appendix

16-item Affective Style Questionnaire, Dutch translation

Concealing/Verbergen
C1 Mensen kunnen meestal niet zien hoe ik me vanbinnen voel.
C2 Ik onderdruk mijn emotionele reacties op gebeurtenissen vaak.
C3 Ik kan mijn gevoelens goed verstoppen.
C4 Mensen merken het meestal niet wanneer ik overstuur ben.
C5 Mensen kunnen het meestal niet aan me zien wanneer ik verdrietig ben.
C6 Ik kan me op zo'n manier gedragen dat mensen het niet aan me zien dat ik van streek ben.
C7 Ik zou emoties gemakkelijk kunnen faken.
C8 Ik kan mijn kwaadheid goed verbergen als het nodig is.
Adjusting/Aanpassen
A1 Ik heb mijn emoties goed onder controle.
A4 Ik kan heel snel kalmeren.
A5 Ik kan een slecht humeur snel achter me laten.
A6 Ik weet exact wat ik moet doen om mezelf in een beter humeur te krijgen.
A7 Ik kan gemakkelijk in een beter humeur geraken.
Tolerating/Verdragen
T2 Het is ok dat mensen zien dat ik van streek ben.
T3 Het is ok om af en toe negatieve emoties (zoals boosheid of verdriet) te voelen.
T5 Er is niks mis met je heel erg emotioneel te voelen.
